# HDAC6 Inhibition Alleviates Ischemia- and Cisplatin-Induced Acute Kidney Injury by Promoting Autophagy

**DOI:** 10.3390/cells11243951

**Published:** 2022-12-07

**Authors:** Lang Shi, Zhixia Song, Chenglong Li, Fangjing Deng, Yao Xia, Jing Huang, Xiongfei Wu, Jiefu Zhu

**Affiliations:** 1 Department of Nephrology, Renmin Hospital of Wuhan University, Wuhan 430064, China; 2 Department of Nephrology, The First Clinical Medical College of Three Gorges University, Center People’s Hospital of Yichang, Yichang 443000, China; 3 Department of Urology, Renmin Hospital of Wuhan University, Wuhan 430064, China; 4 Department of Organ Transplantation, Renmin Hospital of Wuhan University, Wuhan 430064, China

**Keywords:** acute kidney injury, HDAC6, autophagy, renal ischemia/reperfusion, cisplatin

## Abstract

Histone deacetylase (HDAC) 6 exists exclusively in cytoplasm and deacetylates cytoplasmic proteins such as α-tubulin. HDAC6 dysfunction is associated with several pathological conditions in renal disorders, including UUO-induced fibrotic kidneys and rhabdomyolysis-induced nephropathy. However, the role of HDAC6 in ischemic acute kidney injury (AKI) and the mechanism by which HDAC6 inhibition protects tubular cells after AKI remain unclear. In the present study, we observed that HDAC6 was markedly activated in kidneys subjected to ischemia- and cisplatin (cis)-induced AKI treatment. Pharmacological inhibition of HDAC6 alleviated renal impairment and renal tubular damage after ischemia and cisplatin treatment. HDAC6 dysfunction was associated with decreased acetylation of α-tubulin at the residue of lysine 40 and autophagy. HDAC6 inhibition preserved acetyl-α-tubulin-enhanced autophagy flux in AKI and cultured tubular cells. Genetic ablation of the renal tubular (RT) Atg7 gene or pharmacological inhibition of autophagy suppressed the protective effects of HDAC6. Taken together, our study indicates that HDAC6 contributes to ischemia- and cisplatin-induced AKI by inhibiting autophagy and the acetylation of α-tubulin. These results suggest that HDAC6 could be a potential target for ischemic and nephrotoxic AKI.

## 1. Introduction

AKI is a critical and severe clinical disease manifesting as an abrupt or rapid decline in renal filtration function [1,2]. Patients with AKI events have poor long-term outcomes, including increased mortality and comorbidities such as chronic kidney disease [1]. Renal ischemia/reperfusion (IR) injury and nephrotoxic agents remain the major causes of AKI [3,4]. Cisplatin, a widely used chemotherapeutic agent, has notorious side effects on kidneys, thereby limiting its clinical application [5,6,7].

Autophagy is a bulk protein degradation system that may play an important role in maintaining renal hemostasis [8,9,10]. Autophagy is mainly performed by proteins encoded by autophagy-related genes (Atg) including Atg5, Atg6 (beclin1), Atg7 [11], and LC3 (a mammalian homolog of yeast Atg8) [12]. Emerging evidence indicates that autophagy dysregulation participates in the pathophysiology of AKI. For example, Atg7 knockout in proximal tubular cells results in autophagy inhibition and exhibited accelerated renal loss, aggravated renal tissue damage and tubular apoptosis during ischemic AKI [13]. Microtubules participate in the maturation and traffic of autophagosomes, and interruption of microtubules could lead to the accumulation of autophagosomes, resulting in autophagic flux blocking.

HDACs are a group of deacetylating enzymes subdivided into several classes according to their homologous structure [14]. Class I HDACs (HDAC-1, -2, -3, and -8) have ubiquitous tissue distribution and nuclear localization. Class IIa HDACs (HDAC-4, -5, -7, and -9) shuttle between the nucleus and cytoplasm. Class IIb HDACs (HDAC-6 and -10) are distinguished by their two separate catalytic domains. HDAC6 is a unique HDAC that participates in histone acetylation and deacetylation and also targets several nonhistone substrates, such as α-tubulin, heat shock protein 90, and SMAD7 [15,16,17]. HDAC6 can mediate microtubule acetylation by removing acetyl residues in tubulin [17,18], thereby regulating microtubule structure and maintaining microtubule integrity. Deranged HDAC6 activity has also been implicated in various disorders, such as cancer [19], neurodegenerative diseases [20], and kidney diseases [21].

To date, the role of HDAC6 and the underlying mechanisms involved in AKI remain largely unclear. In the present study, we sought to address these issues by utilizing the highly selective HDAC6 inhibitor Tubastatin A (TA) and HDAC6 knockdown in a mouse model of ischemia- and cisplatin-induced AKI.

## 2. Materials and Methods

### 2.1. Antibodies and Reagents

TA was purchased from Selleckchem (Houston, TX, USA). Antibodies in the present study were derived from the following sources: anti-beclin1 (11306-1-AP), anti-ATG7 (10088-2-AP), anti-acetyl Tubulin (Lys40) (66200-1-Ig), anti-Alpha Tubulin (11224-1-AP), and anti-Cyclophilin B (11607-1-AP), and all secondary antibodies for immunoblot analysis were purchased from Proteintech (RoSDont, IL). Anti-p62 (ab24182, Abcam) was from Abcam (Cambridge, UK). Anti-LC3 (NB100-2220) was obtained from Novus Biologicals. Anti-HDAC6 (7612) and anti-cleaved caspase 3 (9664, CST) were purchased from Cell Signaling Technology (Danvers, MA, USA). Cisplatin was obtained from Sigma (St. Louis, MO, USA).

### 2.2. In Vitro Model of Renal IRI and Cisplatin in BUMPT Cell and Examination of Apoptosis

Boston University mouse proximal tubule cells (BUMPT; obtained from Drs. Lieberthal and Shwartz at Boston University) were cultured in 35 mm dishes at a density of 1.0 × 10^5^ cells/dish to reach 70~80% confluence for subsequent experiments. To mimic in vivo renal IRI, cells were treated with 20 μM Carbonyl Cyanide m-Chlorophenylhydrazine (CCCP) (routinely containing Ca2 + KrebsRinger buffer) for 3 h and then recovered for 2 h in the whole medium. To mimic in vivo cisplatin, cells were treated with cisplatin (20 μM) for 24 h and then collected for experiments. To test the effects of autophagy inhibitors, BUMPT cells were first pretreated with 20 μM chloroquine for 1 h, and the inhibitor was added after further treatment with CCCP and recovery for 2 h. Apoptosis was detected using morphological and tdt-mediated dUTP nick-end labeling (TUNEL) staining. Cells with typical apoptotic features were counted to estimate the percentage of apoptosis.

### 2.3. Animals and Treatment

Male C57BL/6 mice (20–23 g) were purchased from the Experimental Animal Center of China Three Gorges University (Yichang, China). Renal tubule (RT)-Atg7-KO mouse model was established by breeding Atg7 flox/flox mice [22] with KSP 1.3-Cre transgenic mice (Jackson lab No. 012237). C57BL/6-Tg (CAG/RFP/EGFP/Map1LC3B) 1 Hill/J autophagy reporter mice were obtained from the Jackson Laboratory (Strain No. 027139). All the mice were kept at a constant temperature (19–21 °C) in a 12 h cycle of light and shade, and each mouse had free access to food and water. To develop mouse renal IR models, we clamped bilateral renal pedicles for 30 min. Sham mice underwent the same surgical procedure without clamping. To develop a mouse model of cisplatin-induced AKI, the animals were injected intraperitoneally with a single dose of cisplatin (20 mg/kg) or normal saline. To study the effect of the HDAC6 inhibitor TA on AKI, TA (70 mg/kg) in 7.5% DMSO was given intraperitoneally immediately after cisplatin injection and then administered daily. The sham group was injected with an equal volume of DMSO as a control. Five mice were used in each group. Mice were killed 24 h after ischemia/reperfusion and 72 h after cisplatin injection. To further validate the effect of TA on autophagy in cisplatin-induced AKI, autophagy reporter mice (CAG/RFP/EGFP/Map1LC3B) were developed in mouse renal ischemia and cisplatin-induced AKI models. Kidney samples were harvested for protein analysis and histological examination. Blood was taken for the measurement of serum creatinine and blood urea nitrogen (BUN). The animal protocol was reviewed and approved by the Institutional Animal Care and Use Committee at Three Gorges University, China.

### 2.4. Renal Function Assay

Renal function was evaluated with serum creatinine and BUN, which were determined using an automatic biochemistry assay (7600p, Hitachi, Japan).

### 2.5. Plasmids and Lentiviral Transduction

For knockdown of HDAC6, target shRNA sequences were subcloned into GV493-shRNA Lentivector. The three shRNA knockdown sequences for HDAC6 were forward: 5′-cgCTGACTACATTGCTGCTTT-3′, 5′-ccTTGCTGGTGGCCGTATTAT-3′, and 5′-tgAGGATGACCCTAGTGTATT-3′ (Genechem, Shanghai, China).

The recombinant lentiviral plasmids were verified by sequencing and co-transfected with pMD2G and pSPAX2 into BUMPT cells to produce recombinant lentiviral. Lentivirus infections were carried out as described previously [23]. Briefly, the cells were seeded in 24-well plates to achieve approximately 70–80% confluence at 24 h, and then the 10% DMEM medium was removed. Cells were transfected with GV493-shRNA Lentivector for 48 h before adding puromycin for screening. Cells were then kept stable in puromycin. The expression efficiency was determined using protein blot methods.

### 2.6. Cell Viability Assay

The cell viability assay was evaluated using the CCK8 Assay Kit. BUMPT cells were seeded at a density of 4 × 10^4^ cells/well in 100 μL of DMEM medium in the 96-well plate overnight, followed by treatment with 20 μM CCCP in regular Ca^2+^ KrebsRinger buffer for 3 h followed by 2 h of recovery in full culture medium or cisplatin (20 μM) for an additional 24 h. A total of 10 μL of CCK-8 was added to each well, and the cells were subsequently incubated at 37 °C for 2 h. Absorbance was measured at 450 nm using the microplate reader.

### 2.7. Analysis of Autophagy Dynamics in CAG-RFP-EGFP-LC3 Autophagy Reporter

The dynamic process of autophagy (autophagosome formation and maturation to au-tolysosomes) was analyzed in CAG-RFP-GFP-LC3 mice as described [24]. This model utilizes the pH sensitivity of EGFP signals, which are quenched at low pH. Therefore, in autophagosome with neutral pH, CAG-RFP-EGFP-LC3 showed green GFP and red RFP fluorescence. However, in autolysosome, the pH value drops to 4–5, leading to the green EGFP fluorescence quenching and the appearance of acid-insensitive red RFP only spots. The number of GFP-LC3 puncta per cell and RFP-LC3 puncta per cell were counted separately using ImageJ. The number of autophagosomes was indicated by GFP dots and the number of autolysosomes was obtained by subtracting GFP dots from RFP dots. The number of autolysosomes was further divided by the total number of RFP dots to indicate the autophagic flux rate.

### 2.8. Histology and TUNEL Staining

For histology, kidney tissue was embedded in paraffin and cut into 4 μm thick sections for hematoxylin and eosin (H&E). Renal tubules in the cortical and outer stripes of the outer medulla with the following histopathological changes were considered as tubular injury: cell lysis, loss of brush border, and cast formation. Tubular injury was examined in a blinded manner and scored by the percentage of injured tubules, as follows: 0, no damage; 1, <25%; 2, 25–50%; 3, 50–75%; 4, >75%. At least 10 randomly selected fields per mouse were scored for quantification, and the mean was used as the tubular injury score. A TUNEL apoptosis detection system (Promega, Madison, WI, USA) was used to detect cell apoptosis, and DAPI was used to stain the nuclei. The slides were examined with fluorescent microscopy, and the TUNEL-positive cells were counted from 10 randomly picked images for each specimen in the outer medulla and kidney cortex region. Data are presented as numbers of positive cells/per field.

### 2.9. Immunohistochemistry (IHC) and Immunofluorescence

Renal tissues were fixed in 4.5% buffered formalin, dehydrated, and embedded in paraffin. After dewaxing, the sections were heated at 95 °C in citric acid solution at pH 6.0 for epitope retrieval, and quenched in 3% H_2_O_2_ to abolish endogenous peroxidase. Then, standard procedure was applied to determine the expression of HDAC6 (1:100), Anti-LC3 antibody (1:200), and Anti-Beclin1 antibody (1:100) in tissue samples. For LC3 and p62 immunofluorescence, the slides were incubated with anti-LC3 and anti-p62 at 4 °C overnight and 1:500 FIFT or Cy3 goat-anti-rabbit/mouse secondary antibody for 1 h at room temperature. For LC3 quantification, 10–20 high magnification (×400) fields were randomly selected from each slide and the number of LC3 puncta per tubule was evaluated using ImageJ software. For p62 quantification, 10–20 high magnification (×400) fields were randomly selected from each slide and the intensity of p62 was measured by the ImageJ software.

### 2.10. Immunoblot Analysis

Cultured cell or kidney tissue lysates were extracted in SDS buffer and protein concentration was measured by BCA Protein Assay Kit (Thermo Scientific, Waltham, MA, USA). Equal amounts of protein (~10 µg for cell lysate, ~50 µg for tissue lysate) were loaded in each lane, separated by SDS-PAGE under reducing conditions, and transferred onto PVDF membrane for standard immunoblot procedure. Following incubation of primary and secondary antibodies, antigens on the blots were revealed with a chemiluminescence kit. ImageJ was used to calculate relative protein expression. The ratio of acetyl α-tubulin and α-tubulin was measured as relative acetyl α-tubulin expression.

### 2.11. Statistical Analysis

Statistical analysis was conducted using the GraphPad Prism 8.3.0 and data were presented as the mean ± SEM. Student’s *t*-test between two groups or one-way ANOVA followed by Tukey’s post hoc test in groups of more than two were used to establish statistical significance. In F (α,β) = γ for ANOVA, α, β, and γ denote degree of freedom for explained variance, degree of freedom for residual variance, and F value, respectively. Correlation analysis was performed using the Pearson correlation statistical analysis. *p* < 0.05 was considered statistically significant.

## 3. Results

### 3.1. HDAC6 Expression Is Increased after Ischemia- and Cisplatin-Induced Acute Kidney Injury

**Figure 1:**
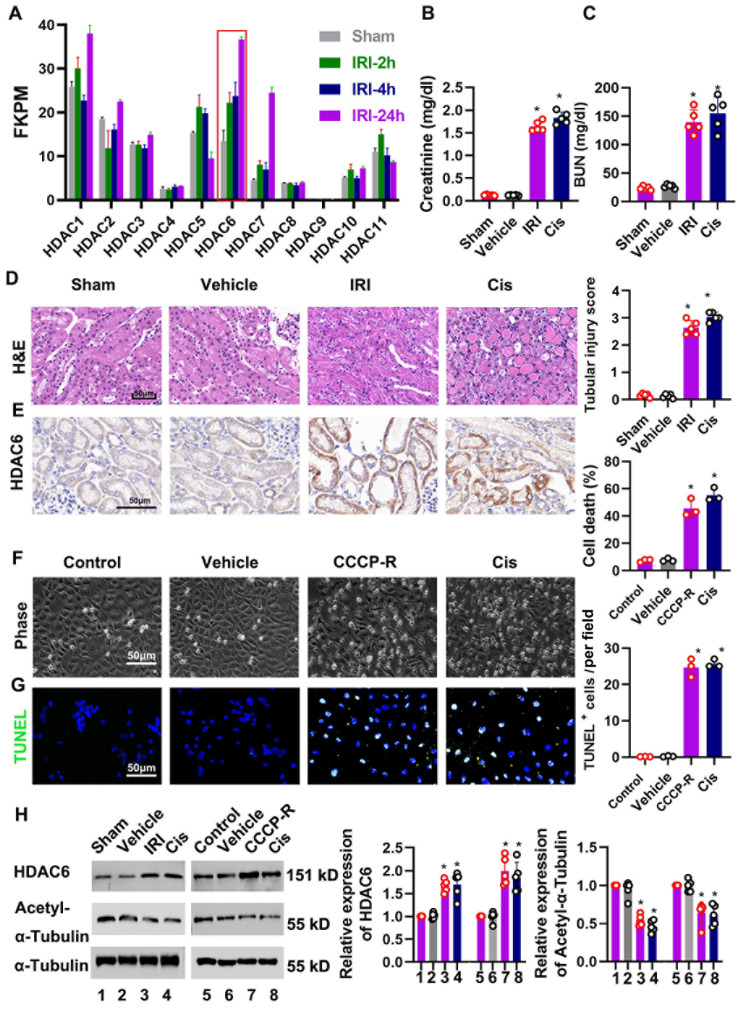
Regulation of HDAC6 in ischemia- and cisplatin-induced AKI. Ischemic renal injury was induced by clamping the bilateral renal particle for 30 min, followed by reperfusion for 24 h. Cisplatin-induced AKI was stimulated by peritoneal injection of cisplatin at a dose of 25 mg/kg, and mice were sacrificed 72 h after injection. (**A**) The transcriptome trend chart of the sham, IRI-2 h, IRI-4 h and IRI 24 d groups using data from GEO (GSE98622). Serum creatinine (**B**) and BUN (**C**) were quantitated as levels of kidney function. *, *p* < 0.05 versus respective sham/vehicle control group (*n* = 5). (**D**) Representative images of H&E staining. Pathological score of tubular damage (**right**). Scale bar = 50 μm. *, *p* < 0.05 versus respective sham/vehicle control group (*n* = 5). (**E**) Representative images of immunohistochemical staining of HDAC6. (**F**) Representative images showing the cell morphology. The percentage of apoptosis was evaluated morphologically (**right**). Scale bar = 50 μm. *, *p* < 0.05 versus respective control/vehicle group (*n* = 3). (**G**) Representative images of TUNEL staining. Quantitative analysis of TUNEL-positive cells (**right**). Scale bar = 50 μm. *, *p* < 0.05 versus respective control/vehicle group (*n* = 3); (**H**) immunoblot of HDAC6 and acetyl-α-tubulin with α-tubulin as internal control. Semi-quantitative analysis (**right**). The ratio of acetyl α-tubulin and α-tubulin was measured as relative acetyl α-tubulin expression. *, *p* < 0.05 versus respective sham/vehicle control group (*n* = 5). FKPM, fragments per kilobase million. (**B**–**D**,**F**,**G**,**H**) Statistical significance was determined by one-way or two-way analysis of variance (ANOVA) followed by Tukey’s post hoc test ((**B**), F (1, 20) = 262.2, *p* < 0.0001; (**C**), F (3, 12) = 58.35, *p* < 0.0001; (**D**), F (3, 12) = 554.9, *p* < 0.0001; (**F**), F (3, 6) = 155.5, *p* < 0.0001; (**G**), F (3, 6) = 260.6, *p* < 0.0001; (**H**), F (7, 32) = 24.31, *p* < 0.0001, F (7, 32) = 31.44, *p* < 0.0001).

HDAC participates in the pathogenesis of AKI by removing acetyl groups from histones and nonhistones [25]. To determine the expression of the HDAC family, we utilized the published RNA sequencing data (GSE98622) and plotted the HDAC expression changes during renal IRI (Figure 1A, Supplementary Table S1). The results show that HDAC6 expression increased continuously within 24 h of reperfusion (Figure 1A). We established bilateral renal ischemia/reperfusion as previously described [26]. Ischemic renal injury was induced by clamping the bilateral renal particle for 30 min, followed by reperfusion for 24 h. Cisplatin-induced AKI was induced by peritoneal injection of cisplatin at a dose of 25 mg/kg, and mice were sacrificed at 72 h after injection. Figure 1B,C show ischemia- and cisplatin-induced renal function. Renal structural changes were evaluated via H&E staining. IR- and cisplatin-induced tubular damages including cast deposition, brush border loss, necrosis, and tubular dilation occurred as expected (Figure 1D). HDAC6 is a specific enzyme responsible for regulating the acetylation process of α-tubulin [17,18]. Worthy of note, HDAC6 expression was increased and acetyl-tubulin was induced after IR and cisplatin treatment (Figure 1E). We further evaluated HDAC6 and acetyl-tubulin in cultured tubular cells during ATP depletion recovery and cisplatin treatment (Figure 1H). BUMPT cells were treated with CCCP for 3 h and allowed to recover for 3 h in full culture media (CCCP-R) to mimic renal IRI in vivo as previously described [27]. CCCP-R and cisplatin treatment induced significant cell death. Consistently, HDAC6 expression increased and ac-tubulin decreased in the in vitro AKI model, suggesting HDAC6 was activated in AKI.

### 3.2. HDAC6 Inhibition Attenuated Ischemia- and Cisplatin-Induced Acute Kidney Injury

**Figure 2:**
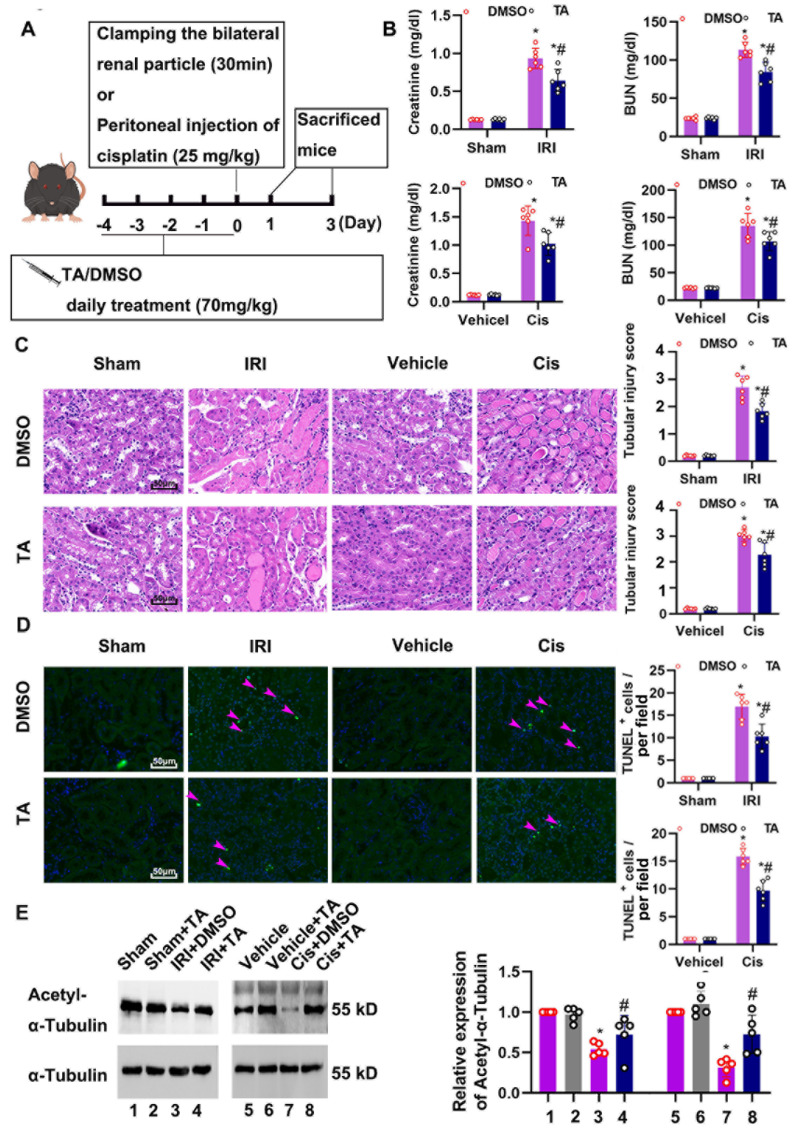
HDAC6 inhibition improved renal dysfunction and pathological changes in the murine model of ischemia-and cisplatin-induced AKI. (**A**) Schematic diagram of the experimental procedure. (**B**) Serum creatinine and BUN were quantitated as levels of kidney function. (**C**) Representative images of H&E staining. Scale bar = 50 μm. Pathological score of tubular damage (**right**). (**D**) TUNEL staining of outer medulla tissue and semi-quantification analysis. Scale bar = 50 μm. (**E**) Immunoblot of acetyl-α-tubulin and semi-quantitative analysis (**right**). *, *p* < 0.05 versus respective sham/vehicle control group. #, *p* < 0.05 versus DMSO-treated IRI or Cis group. (*n* = 5). (**B**–**E**) Statistical significance was determined by one-way or two-way analysis of variance (ANOVA) followed by Tukey’s post hoc test (**B**), F (3, 12) = 441.9, *p* < 0.0001; F (1, 20) = 510.1, *p* < 0.0001; F (1, 20) = 273.2, *p* < 0.0001; F (1, 20) = 299.8, *p* < 0.0001; (**C**) F (1, 20) = 402.3, *p* < 0.0001, F (1, 20) = 547.5, *p* < 0.0001 (**D**) F (1, 20) = 253.0, *p* < 0.0001; F (1, 20) = 583.3, *p* < 0.0001 (**E**), F (7, 32) = 17.85, *p* < 0.0001).

We then examined the role of HDAC6 in ischemia- and cisplatin-induced AKI. HDAC6 inhibitor (Tubastatin A, TA) was administrated 4 consecutive days before IRI and cisplatin treatment. IRI and cisplatin treatment suppressed renal acetyl-tubulin expression, indicating the activation of HDAC6 (Figure 1H). Importantly, HDAC6 inhibition significantly lessened IRI- and cisplatin-induced renal impairment, manifesting as increased SCr and BUN levels (Figure 2B). Consistently, less structural damage was seen in the HDAC6-inhibitor-treated group (Figure 2C). Furthermore, there were fewer TUNEL-positive signals in the HDAC6 inhibition group compared to the vehicle-treated group (Figure 2D). TA administration significantly preserved acetyl-tubulin, indicating the efficiency of TA (Figure 2E).

### 3.3. HDAC6 Inhibition Ameliorated CCCP-and Cisplatin-Induced Tubular Cell Death

**Figure 3:**
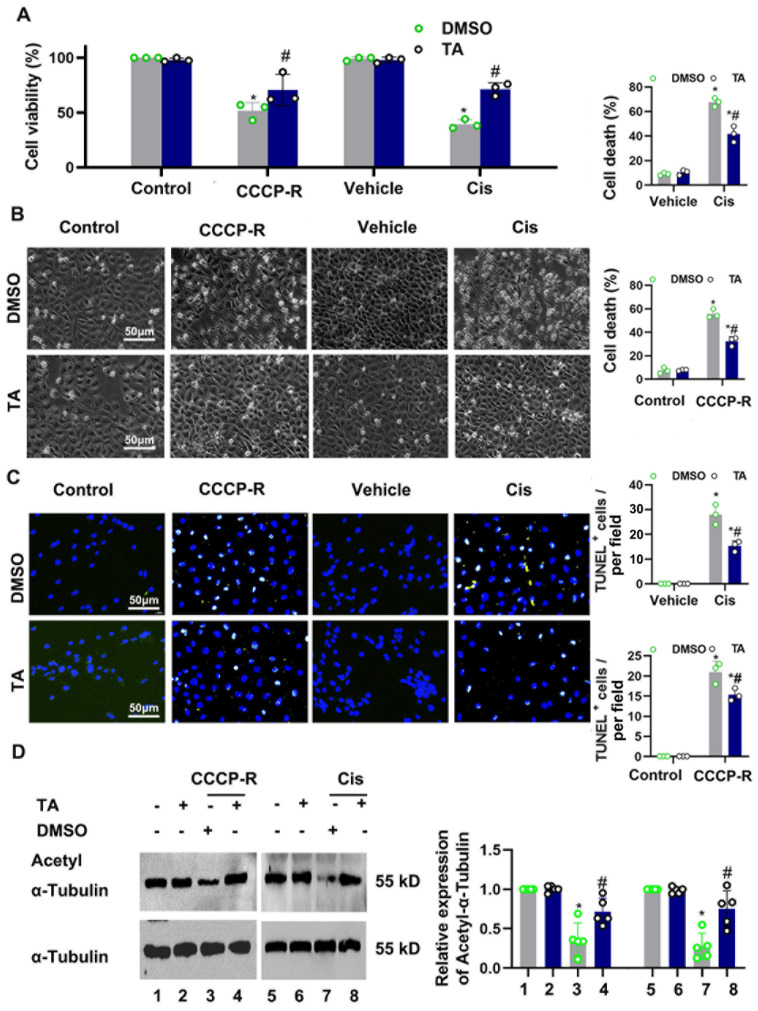
HDAC6 inhibition ameliorated CCCP-R and cisplatin-induced tubular cell death. (**A**) The BUMPT cells’ viability was determined using a CCK-8 assay. (**B**) Representative images showing the cell morphology. Bars = 50 μm. The percentage of apoptosis was evaluated morphologically (**right**). (**C**) Representative images of TUNEL staining. Scale bar = 50 μm. Quantitative analysis of TUNEL-positive cells (**right**). (**D**) Immunoblot of acetyl-α-tubulin and α-tubulin. Semi-quantitative analysis (**right**). Data are expressed as mean ± SD (*n* = 3–5). *, *p* < 0.05 versus respective control group. #, *p* < 0.05 versus DMSO-treated ATP depletion or Cis group. (*n* = 3–5). (**A**–**D**) Statistical significance was determined by one-way or two-way analysis of variance (ANOVA) followed by Tukey’s post hoc test ((**A**), F (3, 16) = 82.65, *p* < 0.0001; (**B**) F (1, 8) = 405.0, *p* < 0.0001, F (1, 8) = 452.5, *p* < 0.0001 (**C**) F (1, 8) = 274.5, *p* < 0.0001; F (1, 8) = 419.7, *p* < 0.0001 (**D**), F (7, 32) = 22.80, *p* < 0.0001).

Proximal tubular cells are vulnerable to ischemia and nephrological toxins such as cisplatin. We next investigated HDAC6 in cultured tubular cells. BUMPTs were pretreated with TA (10 μM) or the vehicle for two hours, followed by CCCP-R and cisplatin treatment. As seen in Figure 3A, the CCK8 assay showed that TA preserved cellular viability under ATP depletion and cisplatin treatment. The ATP depletion recovery treatment induced cellular death, as detected in bright-field and TUNEL staining (Figure 3B,C). Quantification of cell apoptosis was assessed using morphological changes, as previously described [13]. Quantification analysis showed that cellular death under CCCP-R and cisplatin treatment was suppressed by TA (Figure 3B,C). ATP depletion recovery treatment and cisplatin treatment led to deacetylation of α-tubulin, and TA could recover acetyl-tubulin expression (Figure 3D).

### 3.4. Knocked-Down HDAC6 Ameliorated CCCP-Induced Tubular Cell Death

**Figure 4:**
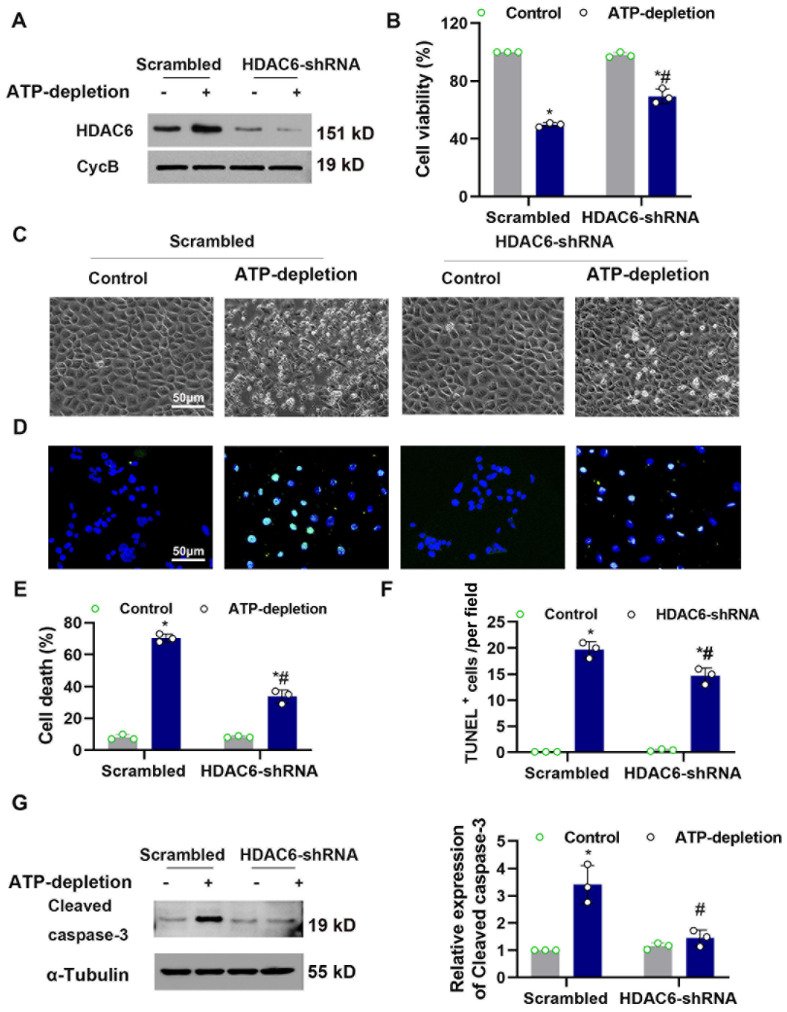
Knocked-down HDAC6 attenuated CCCP-R-induced apoptosis in renal tubular cells. (**A**) Immunoblot showing HDAC6 down-regulation by siRNA in CCCP-treated BUMPT cells. BUMPT cells were stably transfected with lentivirus-containing small interfering RNAs (siRNAs) or scramble siRNA. The whole-cell lysates were evaluated for HDAC6 by immunoblotting with cyclophilin B as the internal control. (**B**) The BUMPT cells’ viability was determined using a CCK-8 assay. (**C**) Representative images showing the cell morphology. Bars = 50 μm. The percentage of apoptosis was evaluated morphologically. (**D**) Representative images of TUNEL staining. Scale bars = 50 μm. (**E**) The percentage of apoptosis was evaluated morphologically. (**F**) Quantitative analysis of TUNEL-positive cells. (**G**) Immunoblot of cleaved caspase-3. Semi-quantitative analysis (**right**). Data are expressed as mean ± SD (*n* = 3). *, *p* < 0.05 versus respective control group. #, *p* < 0.05 versus scramble-treated ATP-depletion or Cis group. (*n* = 3). (**B**,**E**–**G**) Statistical significance was determined by two-way analysis of variance (ANOVA) followed by Tukey’s post hoc test ((**B**), F (1, 8) = 29.25, *p* = 0.0006 (**E**) F (1, 8) = 853.9, *p* < 0.0001 (**F**) F (1, 8) = 749.0, *p* < 0.0001; (**G**), F (1, 8) = 27.98 *p* = 0.0007).

We next investigated the role of HDAC6 in CCCP-induced tubular cell death. HDAC6 stable knockdown cells were generated through lentivirus transduction by short hairpin RNA sequences. Western blot was used to detect HDAC6 expression. As expected, the knockdown approach dramatically reduced the protein expression of HDAC6 in the shRNA cells (Figure 4A). The CCK8 assay showed that HDAC6 shRNA improved cellular viability during CCCP-R (Figure 4B). BUMPTs were transfected with HDAC6 shRNA or NC and were subjected to CCCP-R treatment. As seen in Figure 4C,D, ATP depletion recovery treatment induced cellular death, as detected by bright-field and TUNEL staining. Quantification analysis cellular death, TUNEL staining and cleaved caspase-3 showed that HDAC6 knockdown suppressed cellular injury under CCCP-R treatment (Figure 4E–G). 

### 3.5. HDAC6 Inhibition Promotes Autophagy in Ischemia- and Cisplatin-Induced AKI

**Figure 5:**
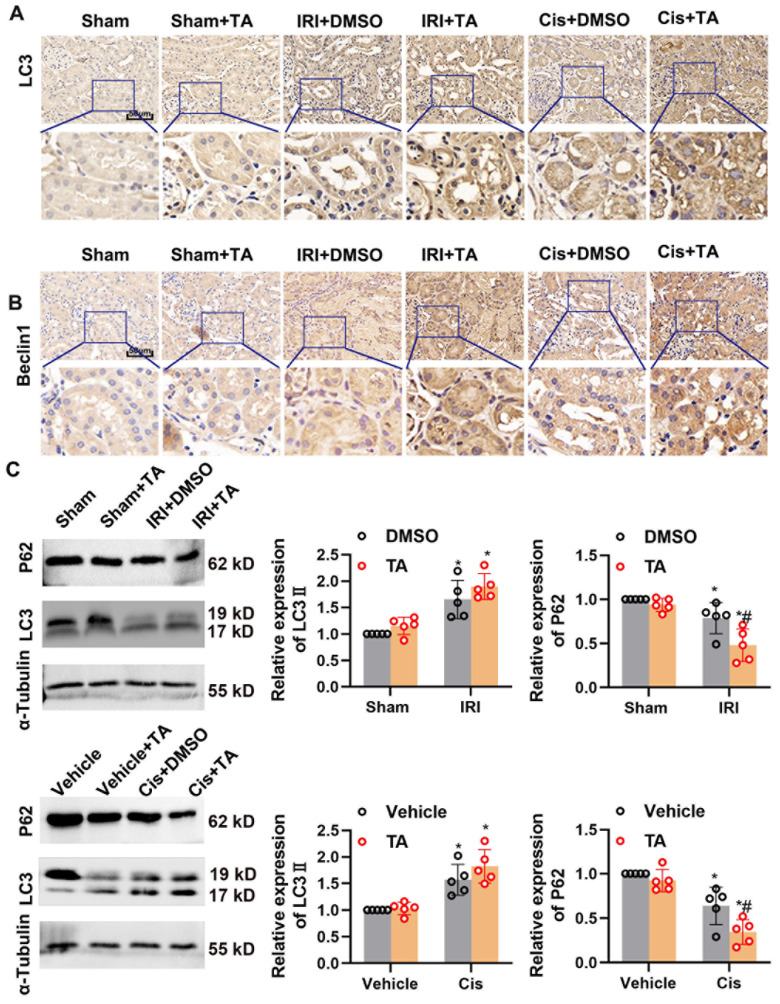
HDAC6 inhibition promotes autophagy in ischemia- and cisplatin-induced AKI. (**A**) Representative images of immunohistochemical staining of LC3. Scale bar = 50 μm. (**B**) Representative images of immunohistochemical staining of Beclin1. Scale bar = 50 μm. (**C**) Immunoblot of P62 and LC3. Semi-quantitative analysis (**right**). *, *p* < 0.05 versus respective sham/vehicle control group. #, *p* < 0.05 versus DMSO/Vehicle-treated IRI or Cis group. (*n* = 5). Statistical significance was determined by two-way analysis of variance (ANOVA) followed by Tukey’s post hoc test (LC3II F (1, 16) = 44.58, *p* < 0.0001, LC3II F (1, 16) = 46.30, *p* < 0.0001; p62 F (1, 16) = 32.00, *p* < 0.0001; p62 F (1, 16) = 56.06, *p* < 0.0001).

**Figure 6:**
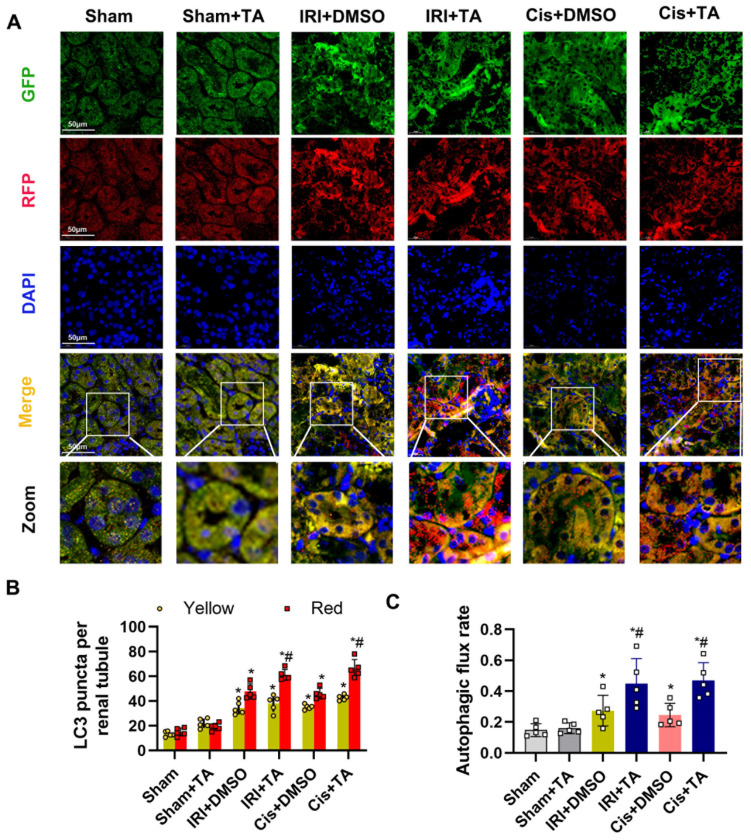
HDAC6 inhibition promoted autophagic flux in autophagy reporter mice. (**A**) Representative images of GFP-LC3 and RFP-LC3 fluorescence staining. Scale bar = 50 μm. (**B**) Quantification of the numbers of autophagosomes and autolysosomes per proximal tubule. (**C**) Quantification of autophagic flux rate. Data are expressed as mean ± SD. *, *p* < 0.05, versus respective control or sham group. #, *p* < 0.05, versus DMSO-treated ATP depletion or Cis group. Statistical significance was determined by two-way analysis of variance (ANOVA) followed by Tukey’s post hoc test (**B**) F (5, 48) = 124.3, *p* < 0.0001; (**C**) F (5, 20) = 8.410, *p* = 0.0002).

We next investigated the mechanism underlying the effects of HDAC6 during ischemic challenges. HDAC6 has been reported to be involved in autophagy regulation in podocytes [28] and proximal tubular cells [29]. Atg7 and Beclin-1 are two conjugation system proteins in autophagosome formation [30]. IRI activation of autophagy manifested as the increased level of LC3 and beclin1 expression in tubular epithelial cells detected by IHC (Figure 5A,B). Immunoblot analysis indicated that p62, a substrate produced during autophagic degradation, was further reduced under TA treatment (Figure 5C). We further examined the autophagic flux in CAG-RFP-EGFP-LC3 autophagy reporter mice. As shown in Figure 6A, renal tubules in control kidneys had few GFP and RFP puncta. IR and cisplatin treatment led to increases in the numbers of both GFP and RFP puncta in tubules, which was further enhanced by TA treatment. We further measured the numbers of yellow (the merge of GFP and RFP) and red puncta and calculated autophagic flux rate in vivo. Quantification analysis demonstrated that TA enhanced the red-only signal and promoted autophagy flux rate during ischemic- and cisplatin-induced AKI in renal tissues (Figure 6B,C). These results together indicate TA further activated autophagy flux in IRI treatment.

### 3.6. Chloroquine Suppresses the Protective Effects of HDAC6 Inhibition on Cellular Viability during CCCP Treatment

**Figure 7:**
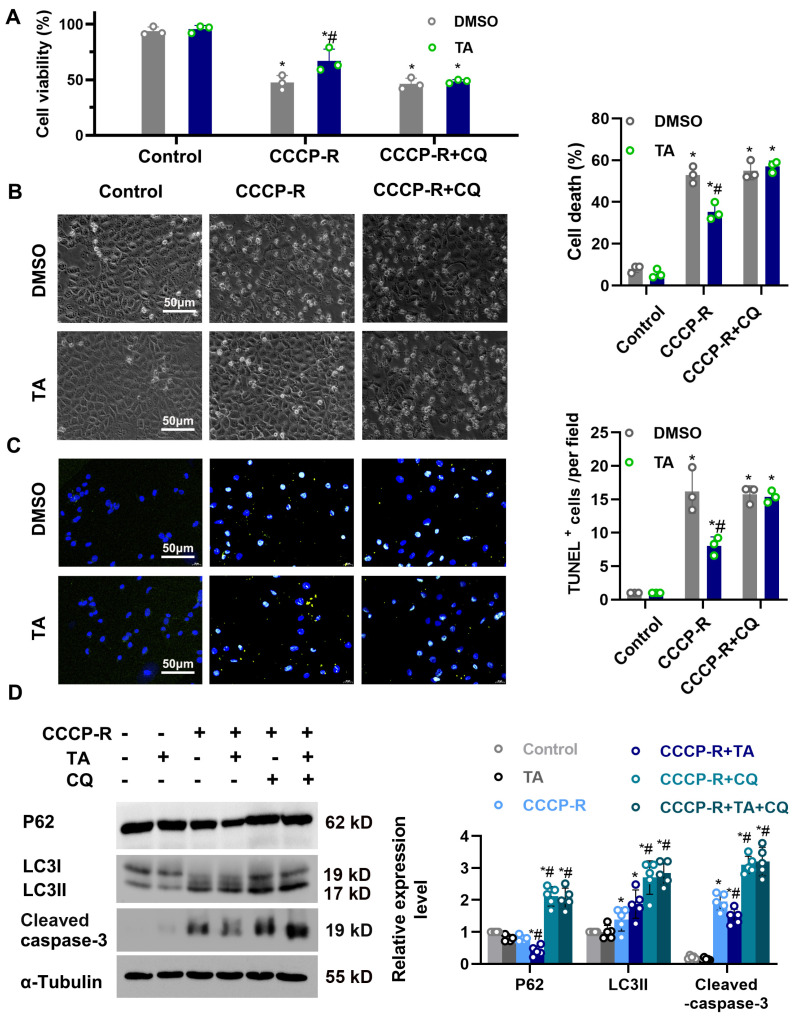
CQ suppressed the protective effects of TA on cellular viability during CCCP treatment. (**A**) The BUMPT cells’ viability was determined using a CCK-8 assay. (**B**) Representative images show the cell morphology. Bars = 50 μm. The percentage of apoptosis was evaluated morphologically (**right**). (**C**) Representative images of TUNEL staining. Scale bar = 50 μm. Quantitative analysis of TUNEL-positive cells (**right**). (**D**) Immunoblot of P62, LC3 and Cleaved caspase-3. Semi-quantitative analysis (**right**). *, *p* < 0.05 versus respective control group. #, *p* < 0.05 versus DMSO-treated ATP depletion or Cis group (*n* = 3–5). (**A**–**D**) Statistical significance was determined by two-way analysis of variance (ANOVA) followed by Tukey’s post hoc test (**A**), F (2, 12) = 107.6, *p* < 0.0001 (**B**) F (2, 12) = 355.6, *p* < 0.0001 (**C**) F (2, 12) = 139.5, *p* < 0.0001; (**D**) p62, F (5, 24) = 63.53, *p* < 0.0001; LC3II F (5, 24) = 25.73, *p* < 0.0001; Cleaved caspase-3, F (5, 24) = 133.2, *p* < 0.0001).

We investigated whether autophagy mediated the effect of HDCA6 in ATP depletion treatment. BUMPTs were pretreated with TA (10 μM) or the vehicle in the presence or absence of chloroquine (CQ) for two hours, followed by CCCP-R treatment. TA reduced tubular apoptosis and improved cellular viability during CCCP-R treatment, effects which could be blocked by CQ (Figure 7A–D). CQ blocked the flux of autophagy and caused autophagy protein accumulation (Figure 7D). Autophagy activation by TA during CCCP-R treatment was also blocked by CQ (Figure 7D).

### 3.7. Ischemia-Induced Autophagy Is Inhibited in Renal Tubules in RT-Atg7-KO Mice

**Figure 8:**
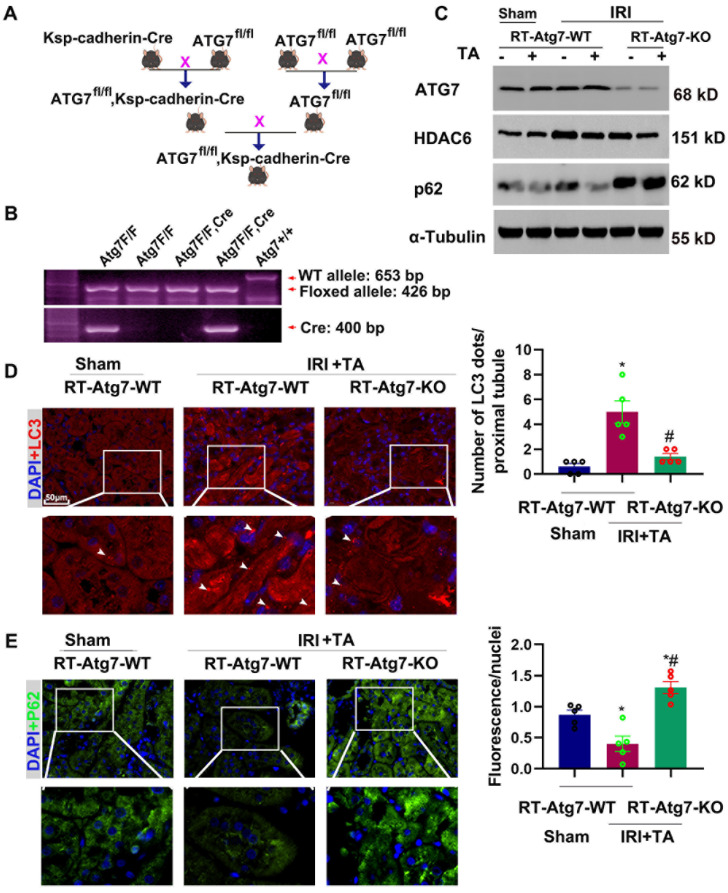
Ischemia-induced autophagy is inhibited in renal tubules in RT-Atg7-KO mice. (**A**) Breeding protocol for generating RT-Atg7-KO mice. Male littermate mice 8–9 weeks old were used for experiments after genotypes were confirmed. (**B**) Representative images of PCR-based genotyping. Genomic DNA was extracted from tail biopsy and amplified to detect wild-type (WT) and floxed alleles of Atg7 and Ksp-cadherin-Cre allele as indicated. (**C**) Whole-tissue lysate of kidney cortex was collected from RT-Atg7-KO and wild-type (RT-Atg7-WT) littermate mice for immunoblot analysis of Atg7, HDAC6, p62, and α-tubulin. (**D**) Representative images of immunofluorescence staining of LC3. Arrows point to LC3 dots. Quantification of LC3 dots in individual tubule of ischemia-treated wild-type and RT-Atg7-KO groups (**right**). (**E**) Representative images of immunofluorescence staining of P62. Semi-quantitative analysis (**right**). *, *p* < 0.05 versus sham group. #, *p* < 0.05 versus RT-Atg7-WT IRI group (*n* = 5). (**D**,**E**) Statistical significance was determined by two-way analysis of variance (ANOVA) followed by Tukey’s post hoc test (**D**), F (2, 12) = 17.91, *p* = 0.0002, (**E**) F (2, 12) = 20.46, *p* = 0.0001).

**Figure 9:**
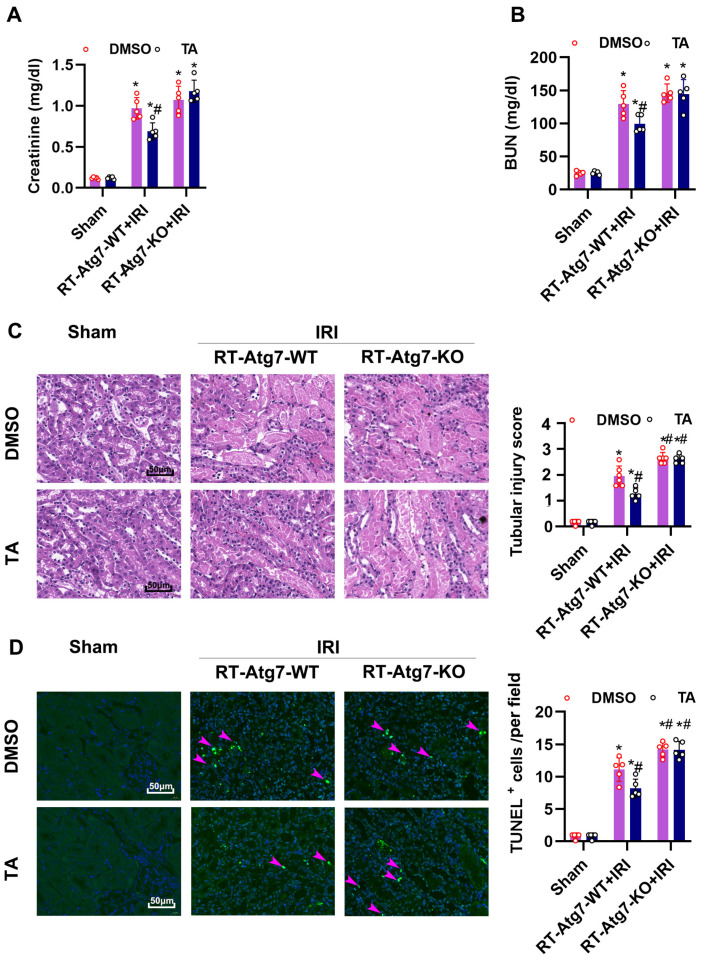
Atg7 depletion in renal tubule suppressed the protective effects of HDAC6 inhibition on renal IRI. (**A**) Serum creatinine and (**B**) BUN were quantitated as levels of kidney function. (**C**) Representative images of H&E staining. Scale bar = 50 μm. Pathological score of tubular damage (**right**). (**D**) TUNEL staining of outer medulla tissue and semi-quantification analysis. Scale bar = 50 μm. *, *p* < 0.05 versus respective sham group. #, *p* < 0.05 versus DMSO-treated IRI group (*n* = 5). (**A**–**D**) Statistical significance was determined by two-way analysis of variance (ANOVA) followed by Tukey’s post hoc test (**A**), F (2, 24) = 216.5, *p* < 0.0001; (**B**) F (2, 24) = 187.4, *p* < 0.0001; (**C**), F (2, 26) = 1122, *p* < 0.0001; (**D**), F (2, 24) = 177.3, *p* < 0.0001).

ATG7 is an essential protein for the induction of autophagy, and the absence of ATG7 leads to a decrease in autophagosome formation [13,30]. To further define the role of TA and tubular cell autophagy in AKI, we established a conditional knockout mouse model, in which Atg7 was deleted specifically from renal tubules in kidneys (Figure 8A,B). Immunoblot analysis confirmed that Atg7 expression was reduced in RT-Atg7-KO mice, compared with their wild-type littermates (Figure 8C). In immunofluorescence and immunoblot analyses, TA led to LC3B-positive puncta accumulation and p62 degradation in renal tubule during IRI in PT-Atg7 WT mice. In contrast, the number of LC3B-II puncta were largely decreased and p62 was accumulated in RT-Atg7-KO kidneys, indicating TA-induced autophagy flux was suppressed in renal tubular cells in RT-Atg7-KO mice (Figure 8C–E). We next tested the effect of TA in renal tubular cells in RT-Atg7-KO. RT-Atg7-KO and RT-Atg7-WT were administrated TA or Vehicle for 4 consecutive days before IRI treatment. With vehicle treatment Atg7 KO mice were more susceptible to IRI than RT-Atg7-WT mice (Figure 9A–D), consistent with previous studies [13]. TA alleviated renal impairment, tubular damages, and TUNEL-positive staining in RT-Atg7-WT mice (Figure 9A–D). However, this protective effect of TA was lost in RT-Atg7-WT mice (Figure 9A–D). These results together indicate that autophagy mediated the protective effects of HDAC6 inhibition in ischemic AKI.

## 4. Discussion

The HDAC family is involved in multiple pathophysiological processes. In this study, we demonstrated that HDAC6 was activated and acetyl-α-tubulin was decreased in ischemia- and cisplatin-induced AKI models. HDAC6 inhibition by TA significantly protected against AKI in animal experiments and cultured tubular cells. The protective effects of TA during AKI were blocked in Atg7 conditional knockout mice. Together, these results demonstrate that HDAC6 inhibition attenuated AKI via the preservation of acetyl-α-tubulin and upregulation of autophagy flux.

Several subtypes of HDAC, including HDAC1, HDAC2, HDAC3, HDAC4, and HDAC9 are involved in the progression of AKI [25]. Further research suggests that trichostatin, a non-selective HDAC inhibitor, has renal-protective effects in transplantation-associated IR [31]. For example, Liu et al. reported that HDAC3 inhibition attenuated cisplatin-induced nephropathy by activating autophagy via AMPK [24]. Xiang et al. reported that the HDAC3 inhibitor alleviated transplantation-induced injury [32]. HDAC6 is the best-characterized class IIb deacetylase and has unique substrate specificity for nonhistone proteins [33]. HDAC6 activation has been reported in cisplatin- and rhabdomyolysis-induced nephropathy [34] in previous studies, and inhibition of HDAC6 alleviated renal damage in these models. Inhibition of HDAC6 protects neurons in ischemic stroke [35]. The role of HDAC6 in renal IRI remains unknown. In the current study, IRI and ATP depletion recovery induced HDAC6 activation. HDAC6 inhibition by TA alleviated cisplatin- and ischemia-induced renal injury.

Autophagy maintains cell homeostasis and survival during initial injuries, although it promotes fibrosis and fibroblast activation during the repairing and remodeling stage [30]. Livingston et al. demonstrated that Atg7 KO aggravated cisplatin and ischemic AKI [13,36]. CQ-inhibited autophagy flux aggravated LPS-induced AKI [37]. In line with these observations, our results confirm that RT-Atg7-KO aggravated ischemia- and cisplatin-induced AKI. The mechanisms underlying HDAC6 inhibition remain unclear, although they may be related to autophagy [29], inflammation [38], or endoplasmic reticulum stress [34]. We demonstrated HDAC6 inhibition recovered acetyl-α-tubulin expression, thus contributing to autophagic flux in autophagy reporter mice (Figure 6). In support of this hypothesis, the protective effects of TA were blocked in RT-Atg7-KO mice, suggesting that autophagy mediated the protective effects of HDAC6 inhibition.

Autophagic flux refers to the complete process of autophagy, beginning with the formation of autophagosomes that fuse with lysosomes to form the autophagosome. LC3-II is a marker protein of autophagy, and an increase in LC3-II levels indicates either enhanced conversion of LC3I to LC3II or impaired degradation through lysosomes. The p62 protein can bridge the binding of ubiquitinated protein and LC3, and promote the clearance of target ubiquitinated proteins through autophagic flux. The simultaneous increase in both p62 and LC3 usually indicates the interruption of autophagic flux. We demonstrated that acetyl-α-tubulin was significantly reduced after ischemic and nephrotoxic AKI. Tubulin acetylation has been shown to result in microtubule stabilization, and microtubule structure plays an important role in the interaction of autophagosomes and lysosomes [39,40]. Recent studies have found that hyperacetylation of α-tubulin is required for the stimulation of autophagy [40,41], while acid-environment-induced reduction in α-tubulin acetylation was found to inhibit autophagic activity and induce rat cardiomyocyte injury [42,43]. Acetyl-α-tubulin expression was found to be negatively correlated with HDAC6 activity [17,18]. Recovery of acetyl-α-tubulin by TA preserved acetyl-α-tubulin, promoted p62 degradation and autophagic flux rate in vitro and in vivo conditions, indicating that HDAC6 inhibition preserves autophagy flux for renal tubular cell hemostasis and kidney protection.

Our results indicate that ischemia- and cisplatin-induced AKI led to HDAC6 activation and that, upon induction, α-tubulin deacetylation and blockage of autophagic flux occurred. HDAC6 inhibition by TA retained acetyl-α-tubulin, stimulated autophagy, and protected against AKI, suggesting a potential target for AKI treatment.

## Data Availability

The data that support the findings of this study are available from the corresponding author upon reasonable request.

## Supplementary Materials

The following supporting information can be downloaded at: https://www.mdpi.com/article/10.3390/cells11243951/s1, Table S1, The RNA sequencing data of the HDAC family in renal ischemia reperfusion injury.
